# Inhibition of TGF-β and NOTCH Signaling by Cutaneous Papillomaviruses

**DOI:** 10.3389/fmicb.2018.00389

**Published:** 2018-03-08

**Authors:** Jordan M. Meyers, Miranda Grace, Aayushi Uberoi, Paul F. Lambert, Karl Munger

**Affiliations:** ^1^Program in Virology, Harvard Medical School, Boston, MA, United States; ^2^Department of Developmental, Molecular and Chemical Biology, Tufts University School of Medicine, Boston, MA, United States; ^3^McArdle Laboratory for Cancer Research, Department of Oncology, School of Medicine and Public Health, University of Wisconsin–Madison, Madison, WI, United States

**Keywords:** viral oncogenesis, squamous cell carcinoma, epidermodysplasia verruciformis, keratinocyte differentiation, hit-and-run carcinogenesis

## Abstract

Infections with cutaneous papillomaviruses have been linked to cutaneous squamous cell carcinomas that arise in patients who suffer from a rare genetic disorder, epidermodysplasia verruciformis, or those who have experienced long-term, systemic immunosuppression following organ transplantation. The E6 proteins of the prototypical cutaneous human papillomavirus (HPV) 5 and HPV8 inhibit TGF-β and NOTCH signaling. The *Mus musculus* papillomavirus 1, MmuPV1, infects laboratory mouse strains and causes cutaneous skin warts that can progress to squamous cell carcinomas. MmuPV1 E6 shares biological and biochemical activities with HPV8 E6 including the ability to inhibit TGF-β and NOTCH signaling by binding the SMAD2/SMAD3 and MAML1 transcription factors, respectively. Inhibition of TGF-β and NOTCH signaling is linked to delayed differentiation and sustained proliferation of differentiating keratinocytes. Furthermore, the ability of MmuPV1 E6 to bind MAML1 is necessary for wart and cancer formation in experimentally infected mice. Hence, experimental MmuPV1 infection in mice will be a robust and valuable experimental system to dissect key aspects of cutaneous HPV infection, pathogenesis, and carcinogenesis.

## Introduction

As of January 2018, more than 300 human papillomavirus (HPV) genotypes are listed in the PAVE database ^1^ (Van Doorslaer et al., 2017). HPVs have ∼8 kb circular double stranded DNA genomes, do not generally infect heterologous hosts and exhibit a preference for infecting either mucosal or cutaneous epithelia. Mucosal HPVs are generally transmitted by sexual contact and mucosal infections with α-genus HPVs are the most common sexually transmitted disease with a prevalence of 70 million cases and an annual incidence of 14 million new transmissions in the United States (CDC, 2015). Mucosal HPVs mostly include members of the α-genus. Clinically, α-HPVs can be designated either as “low-risk” or “high-risk.” Low-risk α-HPV infections trigger benign genital warts, whereas high-risk α-HPVs cause intraepithelial lesions that can undergo malignant progression. Almost 100% of cervical carcinoma are caused by high-risk HPVs and infections with these viruses also contribute to a large percentage of anogenital tract as well as oral cancers (Schiffman et al., 2007). Globally, high-risk α-HPVs infections cause approximately 5% of all human cancers. Despite the availability of efficacious prophylactic vaccines for the most abundant high-risk α-HPVs, there will be approximately 4,170 cervical cancer deaths in 2018 in the United States alone, which means that approximately every 2 h one woman will succumb to cervical cancer (Siegel et al., 2018). Because cancers arise years to decades after the initial infection, a significant impact of these prophylactic vaccines on cervical cancer rates is not expected for another 20 years (Frazer, 2004; Schiller and Lowy, 2012). Unfortunately, there are no antiviral compounds to combat HPV infections (Hellner and Munger, 2011).

Cutaneous HPVs include the β-, γ-, μ-, and ν-genera and some α-HPVs also have a preference for infecting cutaneous epithelia. Cutaneous HPV infections are very frequent and have been linked to development of warts, actinic keratosis, keratoacanthoma, psoriasis, and non-melanoma skin cancers. In Australia, up to 24% of 16- to 18-year-olds have skin warts (Kilkenny et al., 1998). The prevalence of actinic keratosis in the United States is ∼20% and resulted in 47 million dermatology visits in 2006 (Del Rosso et al., 2014). Keratoacanthoma are low grade, rapidly growing skin tumors that, unlike squamous cell carcinomas, are derived from cells located in the hair follicle. Cutaneous HPV genomes are readily detected in hair follicle cells harvested by plucking hair, and a high viral load is a predictor of cancer development (Neale et al., 2013). Keratoacanthoma mostly occur in sun-exposed skin in middle aged patients. The incidence has been estimated at approximately 1:1,000 (Chuang et al., 1993). Cutaneous HPVs have also been frequently detected in psoriasis patients (Mahe et al., 2003). In patients suffering from a rare inherited disease, *epidermodysplasia verruciformis* (EV), and in the rapidly increasing population of chronically immune-suppressed individuals, such as organ transplant patients, lesions caused by cutaneous HPV infections can progress to cutaneous squamous cell carcinomas (cSCCs) (Pass et al., 1977; Orth et al., 1978; Neale et al., 2013). Indeed, cSCCs arising in EV patients were the first human tumors that were conclusively linked to HPV infections. More recently, β- and γ-HPV genomes have also been detected at mucosal sites, suggesting the possibility that these viruses may also infect mucosal epithelia (Bottalico et al., 2011; Hampras et al., 2017; Smelov et al., 2017).

The incidence of cSCCs in the general population has been linked to UV exposure and is rising rapidly with more than 700,000 cases and 8,000 deaths yearly in the United States (Alam and Ratner, 2001; Deady et al., 2014). The estimated annual costs for cSCC treatments in the United States are at $3.8 billion (Guy et al., 2015). Whether and how HPVs may be involved in cSCCs development in immunocompetent individuals, however, remains a matter of debate (Feltkamp et al., 2008), even though a recent meta-analysis provided evidence for a modestly increased overall risk of 1.42 (1.18–1.72) for β-HPV infection and cSCC development (Chahoud et al., 2016).

Hence, β-HPV infections are highly associated with cSCC development in EV patients, correlated in the case of cSCCs arising in immune suppressed patients, and only slightly or sporadically associated with cSCCs in immune competent patients. HPV sequences are not retained and expressed in every cSCC cell. This indicates that, unlike what has been shown for high-risk α-HPV-associated cancers, cutaneous HPVs may not be necessary for tumor maintenance. Cutaneous HPV genomes are almost universally detected in asymptomatic patients (Antonsson et al., 2003a; Kohler et al., 2007), but it is not clear whether this is reflective of active and/or persistent infection. Detection of HPV RNA or protein expression may provide a better estimation of the frequency of active HPV infection in non-neoplastic skin (Borgogna et al., 2012, 2014) and it has been debated whether cutaneous HPVs function as carcinogenic drivers, or if they appear as innocuous passengers in cSCCs that arise in immunocompetent individuals (Quint et al., 2015). A recent study with a transgenic mouse model, has provided evidence for the alternative model that some cutaneous HPVs are drivers of cancer initiation but are not necessary for tumor maintenance (Viarisio et al., 2017).

## Carcinogenic Activities of β-HPVs

Because of their isolation from cSCCs in EV patients, much of the early research efforts with the β-HPVs focused on HPV5 and HPV8. Somewhat surprisingly, HPV5 and HPV8 scored as only weakly transforming or non-transforming in standard cell-based assays (Yamashita et al., 1993; Tommasino, 2017). However, experiments with genetically engineered mice where HPV8 sequences were expressed in basal epithelial cells, impressively demonstrated the oncogenic activity of this HPV. While these mice spontaneously develop skin cancer without the requirement of any additional treatment, they also recapitulated the co-carcinogenic effect of UV exposure (reviewed in Howley and Pfister, 2015). These experiments showed that HPV8 E6, E7 and, uniquely, also E2 could each independently contribute to skin cancer formation when ectopically expressed in basal epithelial cells of a transgenic mouse (Schaper et al., 2005; Pfefferle et al., 2008; Marcuzzi et al., 2009).

The β-HPVs are phylogenetically diverse and as of January 2018, 64 β-HPVs are listed in the PAVE database (see text footnote 1; Van Doorslaer et al., 2017). These are further classified into phylogenetic species. HPV5 and HPV8 together with 19 additional HPVs are classified as species 1, there are 22 members classified as species 2, four are species 3, one is species 4, three are species 5, and 13 have not yet been classified by the HPV reference center.

The E6 and E7 proteins of other β-HPVs, including HPV types 20, 38, and 48 also exhibit carcinogenic activities in transgenic mouse models. Mice expressing β1-HPV20 E6 or E7 from keratin K10 promoter and mice with β2-HPV38 E6/E7 expression from a keratin K14 promoter developed skin cancers when exposed to UV. In contrast, mice expressing the β3-HPV49 E6/E7 genes from a keratin 14 promoter were not susceptible to skin carcinogenesis after UV exposure but developed upper digestive tract carcinomas when treated with the chemical carcinogen 4-nitroquinoline 1-oxide (Michel et al., 2006; Viarisio et al., 2011, 2016). Some of these more recently detected β-HPVs also exhibit transforming activities in cell-based assays. Ectopic expression of the β1 HPV24 or HPV36 E6/E7 proteins causes life span extension of primary human foreskin keratinocytes (HFKs), whereas expression of the β2 HPV38 and β3 HPV49 E6/E7 proteins can trigger HFK immortalization (Cornet et al., 2012).

Despite similar genomic organization, there are some differences between the β- and high-risk α-HPVs (McLaughlin-Drubin et al., 2012). Most strikingly, β-HPVs do not encode E5 proteins, which are oncogenic in high-risk α-HPVs (Genther et al., 2003; Maufort et al., 2007, 2010). Moreover, while high-risk α-HPV-associated cervical carcinomas are generally non-productive, cSCCs arising in EV patients are productive infections. Despite these differences, early mechanistic analyses of the oncogenic activities of β-HPVs were modeled after studies with high-risk α-HPV E6 and E7 proteins. Like high-risk α-HPV E6 and E7 proteins, β-HPV E6 and E7s are low molecular weight, cysteine-rich, metal binding proteins of approximately 150 and 100 amino acids, respectively. They lack intrinsic enzymatic activities, are not known to directly bind specific DNA sequences and presumably exert their biological activities by interacting with and functionally modifying cellular regulatory proteins (Munger et al., 2004; Roman and Munger, 2013; Vande Pol and Klingelhutz, 2013). The best studied cellular targets of the high-risk α-HPV E6 and E7 proteins are the TP53 and RB1 tumor suppressors, respectively. The transforming activities of papillomaviruses are a reflection of their life cycles, specifically their need to secure the availability of cellular replication factors in terminally differentiated epithelial cells that would have normally withdrawn from the cell division cycle (Harden and Munger, 2017). Papillomaviruses need to uncouple cell-cycle withdrawal from epithelial differentiation, effectively allowing for unlicensed proliferation in suprabasal keratinocytes. Aberrant DNA synthesis can be sensed by cells and triggers cell abortive responses, which will need to be inhibited by the virus. The strategies that specific papillomaviruses have evolved to enable their productive replication cycles largely determine their oncogenic potential (McLaughlin-Drubin and Munger, 2009; Moody and Laimins, 2010).

The high-risk HPV E7 proteins cause S-phase competence in differentiating cells by targeting the retinoblastoma tumor suppressor, RB1, for degradation. Uncontrolled S-phase entry as a consequence of RB1 inactivation causes activation of the TP53 tumor suppressor, which triggers a transcriptional program to eliminate such rogue cells by apoptosis. This simple, albeit likely not entirely correct, model (Munger et al., 2004; Munger and Jones, 2015) also served as a blueprint for many studies with the EV-associated β-HPVs, even though it became clear very quickly that it was unlikely to apply to these viruses (McLaughlin-Drubin et al., 2012; Meyers and Munger, 2014; Tommasino, 2017). HPV8 E6 does not detectably interact with TP53 or the UBE3A (E6AP) E3 ubiquitin ligase that is necessary for high-risk α-HPV E6 mediated TP53 degradation (Elbel et al., 1997; White et al., 2012a; Brimer et al., 2017). HPV5 and HPV8 E7s weakly bind and do not degrade RB1, similar to the low-risk α-HPV E7s (Yamashita et al., 1993).

All known β-HPV E7 proteins contain canonical LXCXE (L, leucine; C, cysteine; E, glutamate; X, any amino acid)-based binding sites for the retinoblastoma tumor suppressor family of proteins and many have been documented to bind RB1, RBL1 (p107), and RBL2 (p130). Some β-HPV E6 and E7 in fact interfere with RB1 and TP53 stability, reminiscent of cervical cancer associated, high-risk α-HPVs (Caldeira et al., 2003; Accardi et al., 2006; Cornet et al., 2012; White et al., 2014). The interactions described for β-HPV E6 proteins with TP53 are particularly complex as some have been reported to stabilize TP53 (HPV17, HPV38, and HPV92), while others destabilize it (HPV49) and some have been reported to interfere with TP53 activation and/or TP53 mediated activation of certain target genes (HPV5, HPV8, and HPV38) (Cornet et al., 2012; Wallace et al., 2014; White et al., 2014). Whether humans infected with cutaneous HPVs that target RB1 and TP53 similar to the high-risk α-HPVs are at a particularly high risk for developing cSCC, remains to be determined.

Given the fact that most β-HPV-associated tumors arise in sun exposed areas, it was investigated whether these viruses might interfere with UV-induced apoptosis and/or DNA damage repair. Consistent with expectations, several groups reported that β-HPV E6 proteins cause degradation of BAK, a pro-apoptotic BCL2 family member that is released from the mitochondria following UV exposure (Jackson et al., 2000). BAK degradation by β-HPV E6 proteins is predicted to inhibit apoptosis and allow for survival of UV-damaged cells that may have possibly acquired oncogenic mutations (Leverrier et al., 2007; Hufbauer et al., 2015).

Other studies showed that the repair of UV-damaged DNA damage is inhibited in HPV8 E6 expressing cells. HPV5 and HPV8 E6 proteins were reported to bind the EP300/CREBBP acetyltransferases and target them for degradation thereby inhibiting the Ataxia Telangiectasia Mutated (ATM) and Ataxia Telangiectasia and Rad3-Related (ATR) kinases, key sensors, and mediators of double strand DNA break repair. By abrogating ATM/ATR mediated sensing and/or repair of double strand DNA breaks, cellular mutations may accumulate in β-HPV infected cells, thereby driving carcinogenesis (Howie et al., 2011; Wallace et al., 2012; Wendel and Wallace, 2017). These findings support a “hit-and-run” mechanism for β-HPV carcinogenesis where viral gene expression contributes to cancer initiation by increasing the mutational burden caused by UV exposure but is not necessary for tumor maintenance. This would explain why viral gene expression is not maintained in all tumor cells (Arron et al., 2011). Such a model was recently recapitulated with HPV38 transgenic mice (Viarisio et al., 2018). Interestingly, unbiased proteomic analyses of the HPV38 E6 associated proteins provided no evidence for EP300/CREBBP binding to HPV38 E6 (White et al., 2012a), suggesting that inhibition of DNA break repair by β-HPV E6 proteins may not be necessarily linked to EP300/CREBBP degradation.

As mentioned previously, β-HPV genomes have been detected in hair follicles, which contain epithelial stem cells and HPV8 has been reported to increase the number of keratinocytes with stem cell characteristics both in cell- and animal-based model systems (Hufbauer et al., 2013; Lanfredini et al., 2017; Marthaler et al., 2017). Several studies have shown that the TP53 family member and epithelial stem cell marker TP63 is dysregulated in HPV8 E6 expressing keratinocytes. Specifically, higher levels of the amino terminally truncated ΔNp63α and lower levels of full-length TAp63 isoforms were detected in HPV8 E6 expressing human and murine keratinocytes (Lanfredini et al., 2017; Marthaler et al., 2017). NOTCH inhibition decreased TP63 levels in HPV8 E6 expressing undifferentiated keratinocytes, but TP63 expression remained high in differentiating HPV8 E6 expressing keratinocytes, suggesting that there are NOTCH independent pathways that cause deregulated TP63 expression (Meyers et al., 2013). One of these pathways involves down-regulation of microRNA-203 (miR-203) by HPV8 E6 by inhibiting EP300 which in turn affects CCAAT/enhancer-binding protein a (CEBPA) transcription factor activity that controls miR-203 expression. Significantly, up-regulation of miR-203 during calcium-induced differentiation is abrogated in HPV8 E6 expressing keratinocytes (Marthaler et al., 2017). High-level TP63 and low levels of miR-203 were also detected throughout the lesional tissue of an HPV8 positive EV patient (Marthaler et al., 2017). Since EP300 binding is not shared by all β-HPV E6 (White et al., 2012a) it remains to be determined whether this pathway is universally targeted by β-HPV E6 proteins.

## The γ-HPVs; Benign Flora or Dangerous Villains?

As of January 2018, a total of 171 γ-HPVs have been detected, which are classified into 27 different species. The number of species may increase, since 93 γ-HPVs have not yet been classified into species by the HPV reference center (see text footnote 1; Van Doorslaer et al., 2017). The enormous phylogenetic diversity of γ-HPVs is also reflected in their genome organization; members of the γ6 species, for example, do not encode recognizable E6 proteins and some γ-HPVs encode E7 proteins that lack the canonical LXCXE binding site for the RB1 tumor suppressor and the related p107 (RBL1) and p130 (RBL2) proteins. The γ-HPVs were initially isolated from cutaneous epithelia and are often regarded as components of the normal skin viral flora (Antonsson et al., 2003a,b; Forslund, 2007; Chen et al., 2008; Foulongne et al., 2012). More recent studies also detected γ-HPV DNA at mucosal sites (Bottalico et al., 2011; Smelov et al., 2017).

A “deep sequencing” study of human skin cancers and matched normal tissues showed that HPV197, a γ24-HPV, was the most frequently detected HPV genome in cSCCs and was not present in normal skin (Arroyo Muhr et al., 2015). HPV197 could not have been previously detected because the genome is not recognized by the “consensus” PCR primers that are frequently used for HPV analysis of clinical specimens (Arroyo Muhr et al., 2015). To this date, however, there is no evidence that HPV197 sequences are actively expressed in skin cancers and/or that HPV197 or potentially other γ-HPVs contribute to cSCC development in immunosuppressed or immunocompetent individuals.

Analysis of potential cellular targets by affinity purification followed by mass spectrometry (AP/MS) revealed that HPV197 E6 shares many cellular targets with β-HPV E6 proteins, particularly the β1 HPVs that include HPV5 and HPV8 (Grace and Munger, 2017). The HPV197 E7 protein lacks an LXCXE-based RB1 binding site, yet, consistent with earlier work with the γ1 HPV4 E7 protein (Wang et al., 2010), HPV197 E7 interacts with RB1 (Grace and Munger, 2017). Interestingly, however, HPV197 E7 does not bind to RBL1 or RBL2 (Grace and Munger, 2017). The detection of HPV197 DNA in human skin cancers but not normal skin, and the interactions of the E6 and E7 proteins with human tumor suppressor proteins are consistent with the possibility that HPV197 and potentially other γ-HPVs may be associated with skin cancer development. Currently, however, only very few studies have explored the biological activities of γ-HPVs in cell-based or animal models.

## MmuPV1, An Animal Model for Cutaneous HPV Infections

Papillomaviruses are species specific and generally do not infect heterologous hosts (Knipe and Howley, 2013). This has severely hindered the study of PV pathogenesis *in vivo*. Research has also been stymied by the lack of a papillomavirus that naturally infects and replicates in a genetically tractable laboratory animal. The isolation of *Mus musculus* papillomavirus 1, MmuPV1, from skin warts on immunodeficient nude mice promises to revolutionize PV pathogenesis studies (Ingle et al., 2011; Joh et al., 2011). The ability to cause wart formation upon experimental infection with quasivirus or by naked DNA inoculation was originally observed in mouse strains that have T-cell deficiencies or are temporarily immune suppressed (Handisurya et al., 2014; Joh et al., 2016; Uberoi et al., 2016). Lesions arising due to MmuPV1 infection have malignant potential and can progress to cSCCs. Upon UVB-exposure, experimental MmuPV1 infection also causes skin warts that can progress to cSCCs in immunocompetent, standard laboratory mouse strains (Uberoi and Lambert, 2017). Importantly, there is a correlation between immunosuppression due to UV irradiation and onset of MmuPV1 dependent cutaneous papillomas (Uberoi et al., 2016).

MmuPV1 is a member of the Pi genus, which encompasses rodent PVs (Van Doorslaer et al., 2017) and is related to γ- and β-HPVs (Joh et al., 2011). Consistent with these phylogenetic relationships, the MmuPV1 E6 protein has similar cellular targets as the β-HPV8 E6 protein (Meyers et al., 2017). The MmuPV1 E7 protein is related to the γ-HPV197 E7 protein in that it lacks the canonical LXCXE-based RB1 binding site (Joh et al., 2011; Grace and Munger, 2017). Moreover, transcriptomic analyses revealed that similar to cutaneous HPVs, MmuPV1 expresses E6 and E7 from two separate early promoters (Xue et al., 2017).

Because of its ability to infect cutaneous epithelia and cause lesions capable of malignant progression upon UV exposure, MmuPV1 may be an important model for determining the potential roles of β- and some γ-HPVs in skin cancer development.

Interestingly, MmuPV1 can productively infect both cutaneous and mucosal tissues (Cladel et al., 2013, 2016; Hu et al., 2015) and thus may also model aspects of mucosal pathogenesis. In any case, results from infection experiments with mutant MmuPV1 genomes can be combined with infection studies of relevant, genetically engineered mouse models. This will enable studies to define mechanisms of action of viral proteins and to determine how viral targeting of specific cellular signaling pathways contribute to the life cycle, pathogenesis and carcinogenicity of a papillomavirus in a natural host.

## Subversion of TGF-β and Notch Signaling by Cutaneous Papillomaviruses

While inhibition of UV-mediated apoptosis and/or DNA damage sensing/repair provides a satisfying model to mechanistically support a hit-and-run mechanism of cutaneous HPV mediated carcinogenesis, it does not explain how these viruses may cause warts, a necessary precursor to cSCCs. Moreover, studies with MmuPV1 showed that UV-mediated immune suppression rather than mutagenesis was important for pathogenesis and tumor development (Uberoi et al., 2016).

### Inhibition of TGF-β Signaling

**FIGURE 1:**
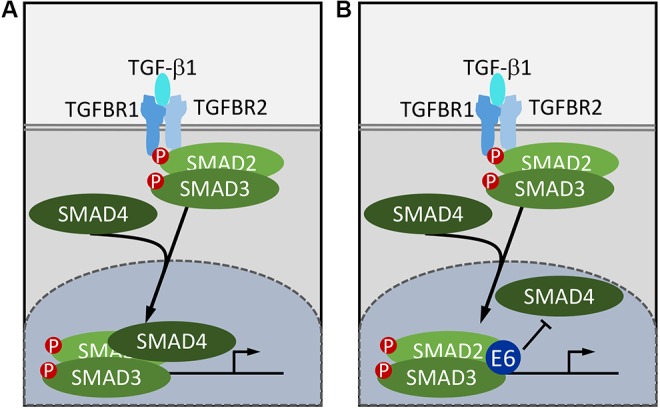
Subversion of TGF-β signaling by HPV8 and MmuPV1 E6. In normal cells, TGF-β binding to the heterotetrameric serine/threonine kinase receptor complex triggers a phosphorylation cascade resulting in phosphorylation of receptor the SMADs, SMAD2, and SMAD3. Phosphorylated receptor SMADs disassociate from the receptor, form a complex with the co-SMAD, SMAD4, and enter the nucleus. The SMAD2/SMAD3/SMAD4 complex then binds to SMAD binding sites to stimulate target gene transcription **(A)**. HPV8 and MmuPV1 E6 proteins bind SMAD2 and SMAD3 and inhibit TGF-β signaling by preventing the formation of the active DNA bound SMAD2/SMAD3/SMAD4 complex **(B)**.

Multiple developmental and cellular processes are regulated by TGF-β signaling. The core components of this signal transduction pathway consist of the ligand, TGF-β, heterotetrameric receptors with serine/threonine kinase activity, receptor associated SMAD (R-SMADs) proteins, and co-activator SMADs (co-SMADs). The tetrameric receptor consists of one dimer of the ligand interacting TGF-β type II receptor (TGFBR2), and one dimer of TGF-β type I receptor (TGFBR1). TGF-β binding triggers a phosphorylation cascade from TGFBR2 to TGFBR1 to R-SMADs (Chaikuad and Bullock, 2016). Activin/Nodal and TGFBR1 receptors phosphorylate the R-SMAD pair SMAD2 and SMAD3, whereas the Bone Morphogenetic Protein (BMP) type I receptors activate the R-SMADs SMAD1, SMAD5, and SMAD8 (Miyazawa et al., 2002). After phosphorylation, R-SMADs complex with the co-SMAD, SMAD4, enter the nucleus, bind specific DNA sequences, and activate target gene transcription (**Figure 1A**). Transcriptional activation of the CDK2 inhibitors p21^CIP1^ (CDKN1A) and p27^KIP1^ (CDKN1B), and the CDK4/CDK6 inhibitor p15^INK4B^ (CDKN2B) have been linked to TGF-β mediated G1 cell cycle arrest (Datto et al., 1995; Reynisdottir et al., 1995). Hence, TGF-β functions as a tumor suppressor in epithelial cells, and TGF-β pathway loss of function mutations are frequent in epithelial tumors (Wang et al., 2000; Qiu et al., 2007; Fleming et al., 2013). Importantly, however, TGF-β signaling also induces expression of factors involved in epithelial to mesenchymal transition (EMT) and causes increased production of matrix metalloproteases, which results in enhanced migration and invasion (Valcourt et al., 2016). This is consistent with an oncogenic activity of TGF-β as was originally discovered in fibroblasts (Roberts et al., 1985). Hence, the NOTCH and TGF-β signaling pathways each have tumor suppressor activities at early stages of carcinogenesis and can function as oncogenes during later stages of carcinogenesis.

The first evidence that cutaneous HPVs inhibit TGF-β signaling was provided by Michel Favre’s group who reported that HPV5 E6 could target SMAD3 for proteasomal degradation (Mendoza et al., 2006). Consistent with this report, AP/MS experiments provided evidence for SMAD2 and SMAD3 association with HPV8 E6 (Meyers et al., 2017). Similarly, the MmuPV1 E6 protein was also shown to bind SMAD2 and SMAD3 (Meyers et al., 2017). Unlike many other papillomavirus E6 binding cellular proteins, SMAD2 and SMAD3 do not contain helical LXXLL (L, leucine; X, any amino acid) motifs (Chen et al., 1998). There is no evidence for SMAD2, SMAD3, or SMAD4 destabilization by HPV8 E6 or MmuPV1 E6 either under basal or TGF-β stimulated conditions (Meyers et al., 2017). Moreover, there is no defect in SMAD2/SMAD3 phosphorylation and nuclear translocation following TGF-β stimulation in HPV8 or MmuPV1 E6 expressing human keratinocytes. The HPV8 and MmuPV1 E6 proteins, however, were shown to reduce SMAD4 association with SMAD2 and SMAD3 after TGF-β stimulation and to inhibit SMAD2/3 and SMAD4 binding to target DNA. Hence cutaneous papillomavirus E6 proteins inhibit TGF-β signaling by blocking productive binding of the SMAD2/3/4 complex to TGF-β responsive promoters (Meyers et al., 2017; **Figure 1B**). It will be interesting to determine whether these E6 proteins can also inhibit signaling by TGF-β related cytokines. Interestingly, TGF-β signaling is also inhibited by α-HPVs and high-risk HPV16 has been reported to inhibit TGF-β through E7 (Pietenpol et al., 1990; Lee et al., 2002) as well as E5 (French et al., 2013), but not through E6. Hence the cutaneous and mucosal HPVs share the ability to inhibit TGF-β signaling although they have evolved different mechanisms.

### Inhibition of NOTCH Signaling

**FIGURE 2:**
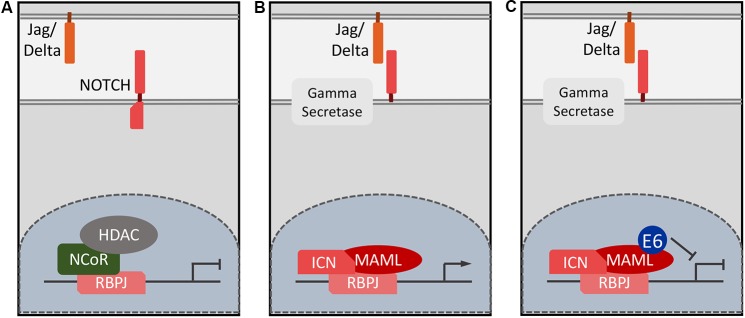
Schematic of NOTCH target gene expression. In the absence of ligand binding, NOTCH responsive genes remain repressed and RBPJ is in a DNA bound, nuclear complex with repressors such as the N-CoR complex **(A)**. Ligand binding leads to NOTCH cleavage and ICN enters the nucleus, displaces repressors and recruits MAML and other co-activators such EP300/CREBBP **(B)**. HPV8 and MmuPV1 E6 proteins inhibit NOTCH signaling by forming a complex with the DNA bound ICN/MAML1 complex thereby preventing formation of a functional transcriptional activator complex **(C)**.

Similar to TGF-β, NOTCH regulates multiple processes during embryogenesis and in the adult organism. Of particular interest to papillomavirus biology, NOTCH signaling drives epithelial differentiation and is required for proper formation of the skin barrier (Rangarajan et al., 2001). NOTCH signaling is triggered through cell-to-cell contact where membrane anchored ligands of the Delta or Jagged family bind to NOTCH family receptors. In humans there are four NOTCH receptors (NOTCH1–4), which exhibit some overlapping functions, though this may be context specific (Kojika and Griffin, 2001). Receptor–ligand binding induces conformational changes in NOTCH that result in a series of proteolytic cleavages ultimately releasing an intracellular fragment of the NOTCH receptor, termed Intracellular NOTCH (ICN; De Strooper et al., 1999). ICN translocates to the nucleus where it associates with Recombination Signal Binding Protein for Immunoglobulin Kappa J Region (RBPJ), which binds to specific DNA sequences. In the absence of ICN, RBPJ is bound to the DNA in association with co-repressors, thus repressing target gene expression (Oswald et al., 2002; **Figure 2A**). ICN displaces these co-repressors and the ICN–RBPJ complex recruits a member of the transcriptional co-activators mastermind-like (MAML) family (Nam et al., 2006). The ICN/RBPJ/MAML complex represents the core NOTCH transcriptional activator complex and MAML then recruits additional co-activators (**Figure 2B**; Saint Just Ribeiro and Wallberg, 2009). Classical NOTCH targets include the Hairy and Enhancer of Split (HES) gene, which belongs to a family of basic helix-loop-helix transcriptional repressors, which are particularly important for lateral inhibition and patterning during development (Iso et al., 2003). NOTCH also regulates other targets such as c-myc (MYC; Weng et al., 2006), cyclin D1 (CCND1; Cohen et al., 2010), and the p21^CIP1^ (CDKN1A) cyclin dependent kinase inhibitor (Rangarajan et al., 2001). Due to the variety of cell types where NOTCH signaling is important, NOTCH target genes are lineage specific and critical NOTCH targets that drive keratinocyte differentiation have not been well-characterized.

Human cSCCs frequently harbor loss of function mutations in the NOTCH pathway, implicating NOTCH activity as tumor suppressive in epithelial cells (Nicolas et al., 2003; Agrawal et al., 2011; Stransky et al., 2011; Wang et al., 2011; South et al., 2014). The tumor suppressive effects of NOTCH in keratinocytes are largely thought to be due to a differentiation-induced block of cell proliferation and the activation of keratinocyte cell death (Zhang et al., 2016; Aster et al., 2017). NOTCH, however, also functions as an oncogenic driver in a number of human tumors, particularly hematopoietic cancers such as T-cell acute lymphoblastic leukemia (T-ALL) where gain of function mutations are commonly observed (Demarest et al., 2008).

MAML1 was originally identified in a yeast-two hybrid screen with HPV16 E6 as a bait (Wu et al., 2000). Like many cellular E6 binding proteins, MAML1 contains LXXLL sequence motifs and several groups detected MAML1 as a cellular interactor of β-HPV E6 proteins in AP/MS experiments (Brimer et al., 2012; Rozenblatt-Rosen et al., 2012; Tan et al., 2012; White et al., 2012a). Similarly, the bovine papillomavirus type 1 (BPV1) E6 protein was also shown to bind MAML1 (Brimer et al., 2012; Tan et al., 2012). Despite the original identification as an HPV16 E6 associated cellular protein (Wu et al., 2000), there is no evidence that mucosal α-HPV E6 proteins detectably associate with MAML1 in mammalian cells (Brimer et al., 2017).

### Biological Consequences of TGF-β and NOTCH Inhibition by Cutaneous Papillomaviruses

Given that the TGF-β and NOTCH pathways are critical in regulating epithelial differentiation and function as tumor suppressors in a variety of epithelial cancers, inhibition of these two pathways by cutaneous papillomavirus E6 proteins may be key to the transforming activities of these viruses.

Consistent with this model, it was shown that keratinocyte differentiation is inhibited in HPV8 E6 expressing epithelial cells. Chromatin immunoprecipitation experiments showed that E6 binds to and inhibits the DNA bound RBPJ/ICN/MAML1 activator complex (Meyers et al., 2013). HPV8 E6 binds to an LXXLL motif within the C-terminal transactivation domain (TAD) 2 of MAML1 (Tan et al., 2012; Meyers et al., 2017). The mechanism of action of TAD2 is unknown; EP300 and related co-activators associate with MAML through its TAD1 domain (Saint Just Ribeiro and Wallberg, 2009; Gerhardt et al., 2014). The MmuPV1 E6 protein also interacts with MAML1 and inhibits NOTCH signaling thereby inhibiting keratinocyte differentiation (Meyers et al., 2017; **Figure 2C**). MAML1 binding defective HPV8 and MmuPV1 E6 mutants were identified using a structure guided approach (Meyers et al., 2017). Of note, many of the HPV8 E6 mutants that were previously used to characterize the biological relevance of the interaction between cutaneous HPV E6 protein and EP300/CREBBP, were found to be MAML1 binding defective, thus further complicating the interpretation of published studies investigating the biological consequences of the EP300/CREBBP interactions with HPV5 and HPV8 E6 (Meyers et al., 2017). Association of E6 with MAML and inhibition of NOTCH signaling is independent of EP300/CREBBP binding, however, since MAML1 binding defective HPV8 E6 mutants retained EP300/CREBBP association and MmuPV1 E6 does not detectably bind EP300/CREBBP (Meyers et al., 2017). Indeed, the notable absence of EP300/CREBBP peptides in AP/MS experiments with MmuPV1 E6 is consistent with a model whereby E6 binding to TAD2 of MAML1 may inhibit expression of NOTCH transcriptional targets by interfering with recruitment or by displacing EP300/CREBBP from MAML1 TAD1. This model, however, awaits experimental validation.

The presence of an intact binding site for MAML1 in MmuPV1 E6 was shown to be necessary for formation of papillomas and carcinomas in experimentally infected mice (Meyers et al., 2017). While these studies cannot definitively exclude the possibility that association of E6 with other LXXLL domain proteins may also contribute to this phenotype, this is unlikely given a recent study that showed that UBE3A and MAML1 are the major LXXLL targets of papillomavirus E6 proteins and, consistent with the published E6/LXXLL structures (Zanier et al., 2013), the various papillomavirus E6 proteins displayed mutually exclusive binding to either UBE3A or MAML1 (Brimer et al., 2017).

Despite the fact that the E6 proteins of mucosal, high-risk α-HPVs have a strong preference for UBE3A binding and do not detectably interact with MAML1 (White et al., 2012a; Brimer et al., 2017), they have also been reported to inhibit NOTCH. HPV16 E6 can inhibit NOTCH signaling indirectly through degradation of the TP53 tumor suppressor (Dotto, 2009) and/or the TAp63β protein (Ben Khalifa et al., 2011). More recent studies showed that NOTCH inhibition is relevant to the biology of these viruses as HPV16 E6 mediated NOTCH inhibition was shown to decrease the commitment of keratinocytes to differentiation (Kranjec et al., 2017).

**FIGURE 3:**
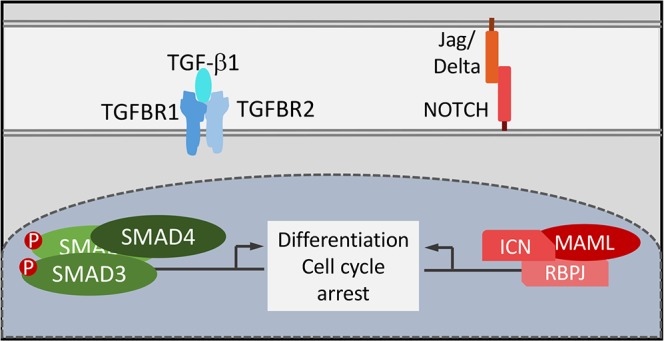
Functional consequences of NOTCH and TGF-β signaling by HPV8 E6 and MmuPV1 E6. TGF-β and NOTCH signaling are critical for cell cycle arrest and keratinocyte differentiation that are tightly coupled in normal squamous epithelial cells. Functional uncoupling of these two processes is a necessary prerequisite for papillomavirus progeny formation in terminally differentiated epithelial cells.

Hence, similar to TGF-β, inhibition of keratinocyte differentiation by targeting NOTCH signaling by cutaneous HPVs is shared with mucosal HPVs but is mediated through different mechanisms. Conceptually this is rather satisfying as papillomaviruses all had to evolve to render normally postmitotic, terminally differentiated epithelial cells conducive for supporting the viral life cycle (**Figure 3**). Interestingly however, different papillomaviruses have evolved mechanistically distinct strategies to solve this problem.

### “Hit and Run” Carcinogenesis: A Consequence of E6 Mediated NOTCH and TGF-β Inhibition?

Hit and run carcinogenesis is a concept that historically has generated some skepticism, also with the authors of this article. The failure to detect viral gene expression in the majority of cancer cells may also imply that β- and γ-HPVs are biologically innocuous passengers that do not drive the carcinogenic process. Given, however, that at least some of these papillomaviruses are carcinogenic in transgenic mouse models and inhibit TGF-β and NOTCH signaling as well as other tumor suppressor pathways, it is more likely that these viruses provide oncogenic hits that drive at least some early aspects of epithelial carcinogenesis. In addition to many other biological activities of cutaneous HPVs, which include stem cell expansion, abrogating apoptosis, sensing and repair of UV-induced double strand DNA breaks, inhibition of the TGF-β and NOTCH tumor suppressors provide plausible oncogenic “hits” during early phases of carcinogenesis. Since the NOTCH and TGF-β pathways are oncogenic at later stages and drive malignant progression (Massague, 2008; Aster et al., 2017) loss of papillomavirus gene expression will re-activate NOTCH and TGF-β. This may drive malignant progression, thereby providing the mechanistic underpinnings for the “run” component of hit and run carcinogenesis.

It is important to note that the β- and γ-HPVs are phylogenetically and biologically diverse (Cornet et al., 2012; Van Doorslaer et al., 2017) and that this diversity is also reflected in the cellular interactomes of their E6 and E7 proteins (White et al., 2012a,b). Hence, findings with one specific β- or γ-HPV type may not be applicable to other HPVs, even within the same genus. The accumulation of UV-induced mutations in a recently published HPV38 transgenic skin cancer model that recapitulates hit-and-run carcinogenesis, for example, is unlikely to be caused through an EP300/CREBBP dependent mechanism as no EP300/CREBBP binding has been detected in unbiased AP/MS studies with HPV38 E6 (White et al., 2012a; Viarisio et al., 2018).

The wide application of AP/MS to define cellular interactomes of papillomavirus proteins has already generated interesting hypotheses, many of which await rigorous validation (White and Howley, 2013; McBride, 2017). Next generation sequencing experiments may detect HPV DNA and/or RNA sequences in unexpected anatomic locations and tentatively link them to unanticipated pathologies (Chen et al., 2017). Such studies may also lead to the identification of HPVs that evaded detection by standard PCR-based techniques. The frequent detection of the novel γ-HPV197 genome in a deep sequencing study of human skin cancers, but not in normal skin, while not implying an etiologic link, should provide at least an impetus to study the biology of γ-HPVs in greater detail (Arroyo Muhr et al., 2015). Given that the HPV197 E7 protein, similar to MmuPV1, lacks an LXCXE-based canonical RB1 binding domain, and binds RB1 through a different sequence, suggest that some aspects of HPV197 E7 can be studied with the MmuPV1 animal model. These and other studies might also reveal whether HPV197, and potentially other γ-HPVs, have oncogenic activities.

## Author Contributions

JM and KM drafted the manuscript. AU, PL, and MG provided editorial and substantive input. KM wrote the final version of the manuscript.

## Conflict of Interest Statement

The authors declare that the research was conducted in the absence of any commercial or financial relationships that could be construed as a potential conflict of interest.

## Footnotes

1 pave.niaid.nih.gov

## References

[B1] Accardi R. Dong W. Smet A. Cui R. Hautefeuille A. Gabet A. S. (2006). Skin human papillomavirus type 38 alters p53 functions by accumulation of deltaNp73. *EMBO Rep.* 7 334–340. 10.1038/sj.embor.7400615 16397624 PMC1456898

[B2] Agrawal N. Frederick M. J. Pickering C. R. Bettegowda C. Chang K. Li R. J. (2011). Exome sequencing of head and neck squamous cell carcinoma reveals inactivating mutations in NOTCH1. *Science* 333 1154–1157. 10.1126/science.1206923 21798897 PMC3162986

[B3] Alam M. Ratner D. (2001). Cutaneous squamous-cell carcinoma. *N. Engl. J. Med.* 344 975–983. 10.1056/NEJM200103293441306 11274625

[B4] Antonsson A. Erfurt C. Hazard K. Holmgren V. Simon M. Kataoka A. (2003a). Prevalence and type spectrum of human papillomaviruses in healthy skin samples collected in three continents. *J. Gen. Virol.* 84 1881–1886. 12810883 10.1099/vir.0.18836-0

[B5] Antonsson A. Karanfilovska S. Lindqvist P. G. Hansson B. G. (2003b). General acquisition of human papillomavirus infections of skin occurs in early infancy. *J. Clin. Microbiol.* 41 2509–2514. 12791874 10.1128/JCM.41.6.2509-2514.2003 PMC156491

[B6] Arron S. T. Ruby J. G. Dybbro E. Ganem D. Derisi J. L. (2011). Transcriptome sequencing demonstrates that human papillomavirus is not active in cutaneous squamous cell carcinoma. *J. Invest. Dermatol.* 131 1745–1753. 10.1038/jid.2011.91 21490616 PMC3136639

[B7] Arroyo Muhr L. S. Hultin E. Bzhalava D. Eklund C. Lagheden C. Ekstrom J. (2015). Human papillomavirus type 197 is commonly present in skin tumors. *Int. J. Cancer* 136 2546–2555. 10.1002/ijc.29325 25388227

[B8] Aster J. C. Pear W. S. Blacklow S. C. (2017). The varied roles of notch in cancer. *Annu. Rev. Pathol.* 12 245–275. 10.1146/annurev-pathol-052016-100127 27959635 PMC5933931

[B9] Ben Khalifa Y. Teissier S. Tan M. K. Phan Q. T. Daynac M. Wong W. Q. (2011). The human papillomavirus E6 oncogene represses a cell adhesion pathway and disrupts focal adhesion through degradation of TAp63beta upon transformation. *PLoS Pathog.* 7:e1002256. 10.1371/journal.ppat.1002256 21980285 PMC3182928

[B10] Borgogna C. Lanfredini S. Peretti A. De Andrea M. Zavattaro E. Colombo E. (2014). Improved detection reveals active beta-papillomavirus infection in skin lesions from kidney transplant recipients. *Mod. Pathol.* 27 1101–1115. 10.1038/modpathol.2013.240 24390217

[B11] Borgogna C. Zavattaro E. De Andrea M. Griffin H. M. Dell’oste V. Azzimonti B. (2012). Characterization of beta papillomavirus E4 expression in tumours from Epidermodysplasia Verruciformis patients and in experimental models. *Virology* 423 195–204. 10.1016/j.virol.2011.11.029 22217391

[B12] Bottalico D. Chen Z. Dunne A. Ostoloza J. Mckinney S. Sun C. (2011). The oral cavity contains abundant known and novel human papillomaviruses from the Betapapillomavirus and Gammapapillomavirus genera. *J. Infect. Dis.* 204 787–792. 10.1093/infdis/jir383 21844305 PMC3156102

[B13] Brimer N. Drews C. M. Vande Pol S. B. (2017). Association of papillomavirus E6 proteins with either MAML1 or E6AP clusters E6 proteins by structure, function, and evolutionary relatedness. *PLoS Pathog.* 13:e1006781. 10.1371/journal.ppat.1006781 29281732 PMC5760104

[B14] Brimer N. Lyons C. Wallberg A. E. Vande Pol S. B. (2012). Cutaneous papillomavirus E6 oncoproteins associate with MAML1 to repress transactivation and NOTCH signaling. *Oncogene* 31 4639–4646. 10.1038/onc.2011.589 22249263 PMC3330202

[B15] Caldeira S. Zehbe I. Accardi R. Malanchi I. Dong W. Giarre M. (2003). The E6 and E7 proteins of the cutaneous human papillomavirus type 38 display transforming properties. *J. Virol.* 77 2195–2206. 10.1128/JVI.77.3.2195-2206.2003 12525654 PMC140944

[B16] CDC (2015). *Epidemiology and Prevention of Vaccine-Preventable Diseases.* Washington, DC: Public Health Foundation.

[B17] Chahoud J. Semaan A. Chen Y. Cao M. Rieber A. G. Rady P. (2016). Association between betα-genus human papillomavirus and cutaneous squamous cell carcinoma in immunocompetent individuals-A meta-analysis. *JAMA Dermatol.* 152 1354–1364. 10.1001/jamadermatol.2015.4530 26720285

[B18] Chaikuad A. Bullock A. N. (2016). Structural basis of intracellular TGF-β signaling: receptors and smads. *Cold Spring Harb. Perspect. Biol.* 8:a022111. 10.1101/cshperspect.a022111 27549117 PMC5088531

[B19] Chen A. A. Gheit T. Stellin M. Lupato V. Spinato G. Fuson R. (2017). Oncogenic DNA viruses found in salivary gland tumors. *Oral Oncol.* 75 106–110. 10.1016/j.oraloncology.2017.11.005 29224806

[B20] Chen A. C. Mcmillan N. A. Antonsson A. (2008). Human papillomavirus type spectrum in normal skin of individuals with or without a history of frequent sun exposure. *J. Gen. Virol.* 89 2891–2897. 10.1099/vir.0.2008/003665-0 18931088

[B21] Chen J. J. Hong Y. Rustamzadeh E. Baleja J. D. Androphy E. J. (1998). Identification of an alpha helical motif sufficient for association with papillomavirus E6. *J. Biol. Chem.* 273 13537–13544. 10.1074/jbc.273.22.13537 9593689

[B22] Chuang T. Y. Reizner G. T. Elpern D. J. Stone J. L. Farmer E. R. (1993). Keratoacanthoma in Kauai, Hawaii. The first documented incidence in a defined population. *Arch. Dermatol.* 129 317–319. 10.1001/archderm.1993.01680240057005 8447667

[B23] Cladel N. M. Budgeon L. R. Balogh K. K. Cooper T. K. Hu J. Christensen N. D. (2016). Mouse papillomavirus MmuPV1 infects oral mucosa and preferentially targets the base of the tongue. *Virology* 488 73–80. 10.1016/j.virol.2015.10.030 26609937 PMC4744521

[B24] Cladel N. M. Budgeon L. R. Cooper T. K. Balogh K. K. Hu J. Christensen N. D. (2013). Secondary infections, expanded tissue tropism, and evidence for malignant potential in immunocompromised mice infected with *Mus musculus* papillomavirus 1 DNA and virus. *J. Virol.* 87 9391–9395. 10.1128/JVI.00777-13 23785210 PMC3754027

[B25] Cohen B. Shimizu M. Izrailit J. Ng N. F. Buchman Y. Pan J. G. (2010). Cyclin D1 is a direct target of JAG1-mediated Notch signaling in breast cancer. *Breast Cancer Res. Treat.* 123 113–124. 10.1007/s10549-009-0621-9 19915977

[B26] Cornet I. Bouvard V. Campo M. S. Thomas M. Banks L. Gissmann L. (2012). Comparative analysis of transforming properties of E6 and E7 from different beta human papillomavirus types. *J. Virol.* 86 2366–2370. 10.1128/JVI.06579-11 22171257 PMC3302372

[B27] Datto M. B. Li Y. Panus J. F. Howe D. J. Xiong Y. Wang X. F. (1995). Transforming growth factor beta induces the cyclin-dependent kinase inhibitor p21 through a p53-independent mechanism. *Proc. Natl. Acad. Sci. U.S.A.* 92 5545–5549. 10.1073/pnas.92.12.5545 7777546 PMC41732

[B28] De Strooper B. Annaert W. Cupers P. Saftig P. Craessaerts K. Mumm J. S. (1999). A presenilin-1-dependent gamma-secretase-like protease mediates release of Notch intracellular domain. *Nature* 398 518–522. 10.1038/19083 10206645

[B29] Deady S. Sharp L. Comber H. (2014). Increasing skin cancer incidence in young, affluent, urban populations: a challenge for prevention. *Br. J. Dermatol.* 171 324–331. 10.1111/bjd.12988 24666396

[B30] Del Rosso J. Q. Kircik L. Goldenberg G. Brian B. (2014). Comprehensive management of actinic keratoses: practical integration of available therapies with a review of a newer treatment approach. *J. Clin. Aesthet. Dermatol.* 7 S2–S12. 25302088 PMC4187997

[B31] Demarest R. M. Ratti F. Capobianco A. J. (2008). It’s T-ALL about Notch. *Oncogene* 27 5082–5091. 10.1038/onc.2008.222 18758476

[B32] Dotto G. P. (2009). Crosstalk of Notch with p53 and p63 in cancer growth control. *Nat. Rev. Cancer* 9 587–595. 10.1038/nrc2675 19609265 PMC6059364

[B33] Elbel M. Carl S. Spaderna S. Iftner T. (1997). A comparative analysis of the interactions of the E6 proteins from cutaneous and genital papillomaviruses with p53 and E6AP in correlation to their transforming potential. *Virology* 239 132–149. 10.1006/viro.1997.8860 9426453

[B34] Feltkamp M. C. De Koning M. N. Bavinck J. N. Ter Schegget J. (2008). Betapapillomaviruses: innocent bystanders or causes of skin cancer. *J. Clin. Virol.* 43 353–360. 10.1016/j.jcv.2008.09.009 18986829

[B35] Fleming N. I. Jorissen R. N. Mouradov D. Christie M. Sakthianandeswaren A. Palmieri M. (2013). SMAD2 SMAD3 and SMAD4 mutations in colorectal cancer. *Cancer Res.* 73 725–735. 10.1158/0008-5472.CAN-12-2706 23139211

[B36] Forslund O. (2007). Genetic diversity of cutaneous human papillomaviruses. *J. Gen. Virol.* 88 2662–2669. 10.1099/vir.0.82911-0 17872517

[B37] Foulongne V. Sauvage V. Hebert C. Dereure O. Cheval J. Gouilh M. A. (2012). Human skin microbiota: high diversity of DNA viruses identified on the human skin by high throughput sequencing. *PLoS One* 7:e38499. 10.1371/journal.pone.0038499 22723863 PMC3378559

[B38] Frazer I. H. (2004). Prevention of cervical cancer through papillomavirus vaccination. *Nat. Rev. Immunol.* 4 46–54. 10.1038/nri1260 14704767

[B39] French D. Belleudi F. Mauro M. V. Mazzetta F. Raffa S. Fabiano V. (2013). Expression of HPV16 E5 down-modulates the TGFbeta signaling pathway. *Mol. Cancer* 12:38. 10.1186/1476-4598-12-38 23651589 PMC3661392

[B40] Genther S. M. Sterling S. Duensing S. Munger K. Sattler C. Lambert P. F. (2003). Quantitative role of the human papillomavirus type 16 E5 gene during the productive stage of the viral life cycle. *J. Virol.* 77 2832–2842. 10.1128/JVI.77.5.2832-2842.2003 12584306 PMC149772

[B41] Gerhardt D. M. Pajcini K. V. D’altri T. Tu L. Jain R. Xu L. (2014). The Notch1 transcriptional activation domain is required for development and reveals a novel role for Notch1 signaling in fetal hematopoietic stem cells. *Genes Dev.* 28 576–593. 10.1101/gad.227496.113 24637115 PMC3967047

[B42] Grace M. Munger K. (2017). Proteomic analysis of the gamma human papillomavirus type 197 E6 and E7 associated cellular proteins. *Virology* 500 71–81. 10.1016/j.virol.2016.10.010 27771561 PMC5127743

[B43] Guy G. P. Jr. Machlin S. R. Ekwueme D. U. Yabroff K. R. (2015). Prevalence and costs of skin cancer treatment in the U.S., 2002-2006 and 2007-2011. *Am. J. Prev. Med.* 48 183–187. 10.1016/j.amepre.2014.08.036 25442229 PMC4603424

[B44] Hampras S. S. Rollison D. E. Giuliano A. R. Mckay-Chopin S. Minoni L. Sereday K. (2017). Prevalence and concordance of cutaneous beta human papillomavirus infection at mucosal and cutaneous sites. *J. Infect. Dis.* 216 92–96. 10.1093/infdis/jix245 28549147 PMC5853403

[B45] Handisurya A. Day P. M. Thompson C. D. Bonelli M. Lowy D. R. Schiller J. T. (2014). Strain-specific properties and T cells regulate the susceptibility to papilloma induction by *Mus musculus* papillomavirus 1. *PLoS Pathog.* 10:e1004314. 10.1371/journal.ppat.1004314 25121947 PMC4133403

[B46] Harden M. E. Munger K. (2017). Human papillomavirus molecular biology. *Mutat. Res. Rev. Mutat. Res.* 772 3–12. 10.1016/j.mrrev.2016.07.002 28528688 PMC5500221

[B47] Hellner K. Munger K. (2011). Human papillomaviruses as therapeutic targets in human cancer. *J. Clin. Oncol.* 29 1785–1794. 10.1200/JCO.2010.28.2186 21220591 PMC3675666

[B48] Howie H. L. Koop J. I. Weese J. Robinson K. Wipf G. Kim L. (2011). Betα-HPV 5 and 8 E6 promote p300 degradation by blocking AKT/p300 association. *PLoS Pathog.* 7:e1002211. 10.1371/journal.ppat.1002211 21901101 PMC3161984

[B49] Howley P. M. Pfister H. J. (2015). Beta genus papillomaviruses and skin cancer. *Virology* 479–480, 290–296. 10.1016/j.virol.2015.02.004 25724416 PMC4424140

[B50] Hu J. Budgeon L. R. Cladel N. M. Balogh K. Myers R. Cooper T. K. (2015). Tracking vaginal, anal and oral infection in a mouse papillomavirus infection model. *J. Gen. Virol.* 96 3554–3565. 10.1099/jgv.0.000295 26399579 PMC4804763

[B51] Hufbauer M. Biddle A. Borgogna C. Gariglio M. Doorbar J. Storey A. (2013). Expression of Betapapillomavirus oncogenes increases the number of keratinocytes with stem cell-like properties. *J. Virol.* 87 12158–12165. 10.1128/JVI.01510-13 24006432 PMC3807911

[B52] Hufbauer M. Cooke J. Van Der Horst G. T. Pfister H. Storey A. Akgul B. (2015). Human papillomavirus mediated inhibition of DNA damage sensing and repair drives skin carcinogenesis. *Mol. Cancer* 14:183. 10.1186/s12943-015-0453-7 26511842 PMC4625724

[B53] Ingle A. Ghim S. Joh J. Chepkoech I. Bennett Jenson A. Sundberg J. P. (2011). Novel laboratory mouse papillomavirus (MusPV) infection. *Vet. Pathol.* 48 500–505. 10.1177/0300985810377186 20685915

[B54] Iso T. Kedes L. Hamamori Y. (2003). HES and HERP families: multiple effectors of the Notch signaling pathway. *J. Cell. Physiol.* 194 237–255. 10.1002/jcp.10208 12548545

[B55] Jackson S. Harwood C. Thomas M. Banks L. Storey A. (2000). Role of Bak in UV-induced apoptosis in skin cancer and abrogation by HPV E6 proteins. *Genes Dev.* 14 3065–3073. 10.1101/gad.182100 11114894 PMC317098

[B56] Joh J. Ghim S. J. Chilton P. M. Sundberg J. P. Park J. Wilcher S. A. (2016). MmuPV1 infection and tumor development of T cell-deficient mice is prevented by passively transferred hyperimmune sera from normal congenic mice immunized with MmuPV1 virus-like particles (VLPs). *Exp. Mol. Pathol.* 100 212–219. 10.1016/j.yexmp.2016.01.003 26778691

[B57] Joh J. Jenson A. B. King W. Proctor M. Ingle A. Sundberg J. P. (2011). Genomic analysis of the first laboratory-mouse papillomavirus. *J. Gen. Virol.* 92 692–698. 10.1099/vir.0.026138-0 21084500

[B58] Kilkenny M. Merlin K. Young R. Marks R. (1998). The prevalence of common skin conditions in Australian school students: 1. Common, plane and plantar viral warts. *Br. J. Dermatol.* 138 840–845. 10.1046/j.1365-2133.1998.02222.x 9666831

[B59] Knipe D. M. Howley P. M. (2013). *Fields Virology.* Philadelphia, PA: Wolters Kluwer.

[B60] Kohler A. Forschner T. Meyer T. Ulrich C. Gottschling M. Stockfleth E. (2007). Multifocal distribution of cutaneous human papillomavirus types in hairs from different skin areas. *Br. J. Dermatol.* 156 1078–1080. 10.1111/j.1365-2133.2007.07809.x 17381450

[B61] Kojika S. Griffin J. D. (2001). Notch receptors and hematopoiesis. *Exp. Hematol.* 29 1041–1052. 10.1016/S0301-472X(01)00676-2 11532344

[B62] Kranjec C. Holleywood C. Libert D. Griffin H. Mahmood R. Isaacson E. (2017). Modulation of basal cell fate during productive and transforming HPV-16 infection is mediated by progressive E6-driven depletion of Notch. *J. Pathol.* 242 448–462. 10.1002/path.4917 28497579 PMC5601300

[B63] Lanfredini S. Olivero C. Borgogna C. Calati F. Powell K. Davies K. J. (2017). HPV8 field cancerization in a transgenic mouse model is due to Lrig1+ keratinocyte stem cell expansion. *J. Invest. Dermatol.* 137 2208–2216. 10.1016/j.jid.2017.04.039 28595997 PMC5613749

[B64] Lee D. K. Kim B. C. Kim I. Y. Cho E. A. Satterwhite D. J. Kim S. J. (2002). The human papilloma virus E7 oncoprotein inhibits transforming growth factor-beta signaling by blocking binding of the Smad complex to its target sequence. *J. Biol. Chem.* 277 38557–38564. 10.1074/jbc.M206786200 12145312

[B65] Leverrier S. Bergamaschi D. Ghali L. Ola A. Warnes G. Akgul B. (2007). Role of HPV E6 proteins in preventing UVB-induced release of pro-apoptotic factors from the mitochondria. *Apoptosis* 12 549–560. 10.1007/s10495-006-0004-1 17195958

[B66] Mahe E. Bodemer C. Descamps V. Mahe I. Crickx B. De Prost Y. (2003). High frequency of detection of human papillomaviruses associated with epidermodysplasia verruciformis in children with psoriasis. *Br. J. Dermatol.* 149 819–825. 10.1046/j.1365-2133.2003.05587.x 14616375

[B67] Marcuzzi G. P. Hufbauer M. Kasper H. U. Weissenborn S. J. Smola S. Pfister H. (2009). Spontaneous tumour development in human papillomavirus type 8 E6 transgenic mice and rapid induction by UV-light exposure and wounding. *J. Gen. Virol.* 90 2855–2864. 10.1099/vir.0.012872-0 19692543

[B68] Marthaler A. M. Podgorska M. Feld P. Fingerle A. Knerr-Rupp K. Grasser F. (2017). Identification of C/EBPalpha as a novel target of the HPV8 E6 protein regulating miR-203 in human keratinocytes. *PLoS Pathog.* 13:e1006406. 10.1371/journal.ppat.1006406 28640877 PMC5481020

[B69] Massague J. (2008). TGFbeta in Cancer. *Cell* 134 215–230. 10.1016/j.cell.2008.07.001 18662538 PMC3512574

[B70] Maufort J. P. Shai A. Pitot H. C. Lambert P. F. (2010). A role for HPV16 E5 in cervical carcinogenesis. *Cancer Res.* 70 2924–2931. 10.1158/0008-5472.CAN-09-3436 20332225 PMC2848882

[B71] Maufort J. P. Williams S. M. Pitot H. C. Lambert P. F. (2007). Human papillomavirus 16 E5 oncogene contributes to two stages of skin carcinogenesis. *Cancer Res.* 67 6106–6112. 10.1158/0008-5472.CAN-07-0921 17616666 PMC2858287

[B72] McBride A. A. (2017). Perspective: the promise of proteomics in the study of oncogenic viruses. *Mol. Cell Proteomics* 16 S65–S74. 10.1074/mcp.O116.065201 28104704 PMC5393395

[B73] McLaughlin-Drubin M. E. Meyers J. Munger K. (2012). Cancer associated human papillomaviruses. *Curr. Opin. Virol.* 2 459–466. 10.1016/j.coviro.2012.05.004 22658985 PMC3422426

[B74] McLaughlin-Drubin M. E. Munger K. (2009). Oncogenic activities of human papillomaviruses. *Virus Res.* 143 195–208. 10.1016/j.virusres.2009.06.008 19540281 PMC2730997

[B75] Mendoza J. A. Jacob Y. Cassonnet P. Favre M. (2006). Human papillomavirus type 5 E6 oncoprotein represses the transforming growth factor beta signaling pathway by binding to SMAD3. *J. Virol.* 80 12420–12424. 10.1128/JVI.02576-05 17020941 PMC1676262

[B76] Meyers J. M. Munger K. (2014). The viral etiology of skin cancer. *J. Invest. Dermatol.* 134 E29–E32. 10.1038/skinbio.2014.6 25302471

[B77] Meyers J. M. Spangle J. M. Munger K. (2013). The human papillomavirus type 8 E6 protein interferes with NOTCH activation during keratinocyte differentiation. *J. Virol.* 87 4762–4767. 10.1128/JVI.02527-12 23365452 PMC3624394

[B78] Meyers J. M. Uberoi A. Grace M. Lambert P. F. Munger K. (2017). Cutaneous HPV8 and MmuPV1 E6 proteins target the NOTCH and TGF-β tumor suppressors to inhibit differentiation and sustain keratinocyte proliferation. *PLoS Pathog.* 13:e1006171. 10.1371/journal.ppat.1006171 28107544 PMC5287491

[B79] Michel A. Kopp-Schneider A. Zentgraf H. Gruber A. D. De Villiers E. M. (2006). E6/E7 expression of human papillomavirus type 20 (HPV-20) and HPV-27 influences proliferation and differentiation of the skin in UV-irradiated SKH-hr1 transgenic mice. *J. Virol.* 80 11153–11164. 10.1128/JVI.00954-06 16971438 PMC1642157

[B80] Miyazawa K. Shinozaki M. Hara T. Furuya T. Miyazono K. (2002). Two major Smad pathways in TGF-β superfamily signalling. *Genes Cells* 7 1191–1204. 10.1046/j.1365-2443.2002.00599.x 12485160

[B81] Moody C. A. Laimins L. A. (2010). Human papillomavirus oncoproteins: pathways to transformation. *Nat. Rev. Cancer* 10 550–560. 10.1038/nrc2886 20592731

[B82] Munger K. Baldwin A. Edwards K. M. Hayakawa H. Nguyen C. L. Owens M. (2004). Mechanisms of human papillomavirus-induced oncogenesis. *J. Virol.* 78 11451–11460. 10.1128/JVI.78.21.11451-11460.2004 15479788 PMC523272

[B83] Munger K. Jones D. L. (2015). Human papillomavirus carcinogenesis: an identity crisis in the retinoblastoma tumor suppressor pathway. *J. Virol.* 89 4708–4711. 10.1128/JVI.03486-14 25673729 PMC4403492

[B84] Nam Y. Sliz P. Song L. Aster J. C. Blacklow S. C. (2006). Structural basis for cooperativity in recruitment of MAML coactivators to Notch transcription complexes. *Cell* 124 973–983. 10.1016/j.cell.2005.12.037 16530044

[B85] Neale R. E. Weissenborn S. Abeni D. Bavinck J. N. Euvrard S. Feltkamp M. C. (2013). Human papillomavirus load in eyebrow hair follicles and risk of cutaneous squamous cell carcinoma. *Cancer Epidemiol. Biomarkers Prev.* 22 719–727. 10.1158/1055-9965.EPI-12-0917-T 23396961

[B86] Nicolas M. Wolfer A. Raj K. Kummer J. A. Mill P. Van Noort M. (2003). Notch1 functions as a tumor suppressor in mouse skin. *Nat. Genet.* 33 416–421. 10.1038/ng1099 12590261

[B87] Orth G. Jablonska S. Favre M. Croissant O. Jarzabek-Chorzelska M. Rzesa G. (1978). Characterization of two types of human papillomaviruses in lesions of epidermodysplasia verruciformis. *Proc. Natl. Acad. Sci. U.S.A.* 75 1537–1541. 10.1073/pnas.75.3.1537 206906 PMC411508

[B88] Oswald F. Kostezka U. Astrahantseff K. Bourteele S. Dillinger K. Zechner U. (2002). SHARP is a novel component of the Notch/RBP-Jkappa signalling pathway. *EMBO J.* 21 5417–5426. 10.1093/emboj/cdf549 12374742 PMC129081

[B89] Pass F. Reissig M. Shah K. V. Eisinger M. Orth G. (1977). Identification of an immunologically distinct papillomavirus from lesions of epidermodysplasia verruciformis. *J. Natl. Cancer Inst.* 59 1107–1112. 10.1093/jnci/59.4.1107 71354

[B90] Pfefferle R. Marcuzzi G. P. Akgul B. Kasper H. U. Schulze F. Haase I. (2008). The human papillomavirus type 8 E2 protein induces skin tumors in transgenic mice. *J. Invest. Dermatol.* 128 2310–2315. 10.1038/jid.2008.73 18401427

[B91] Pietenpol J. A. Stein R. W. Moran E. Yaciuk P. Schlegel R. Lyons R. M. (1990). TGF-β 1 inhibition of c-myc transcription and growth in keratinocytes is abrogated by viral transforming proteins with pRB binding domains. *Cell* 61 777–785. 10.1016/0092-8674(90)90188-K 2140528

[B92] Qiu W. Schonleben F. Li X. Su G. H. (2007). Disruption of transforming growth factor beta-Smad signaling pathway in head and neck squamous cell carcinoma as evidenced by mutations of SMAD2 and SMAD4. *Cancer Lett.* 245 163–170. 10.1016/j.canlet.2006.01.003 16478646 PMC1741856

[B93] Quint K. D. Genders R. E. De Koning M. N. Borgogna C. Gariglio M. Bouwes Bavinck J. N. (2015). Human Beta-papillomavirus infection and keratinocyte carcinomas. *J. Pathol.* 235 342–354. 10.1002/path.4425 25131163

[B94] Rangarajan A. Talora C. Okuyama R. Nicolas M. Mammucari C. Oh H. (2001). Notch signaling is a direct determinant of keratinocyte growth arrest and entry into differentiation. *EMBO J.* 20 3427–3436. 10.1093/emboj/20.13.3427 11432830 PMC125257

[B95] Reynisdottir I. Polyak K. Iavarone A. Massague J. (1995). Kip/Cip and Ink4 Cdk inhibitors cooperate to induce cell cycle arrest in response to TGF-β. *Genes Dev.* 9 1831–1845. 10.1101/gad.9.15.1831 7649471

[B96] Roberts A. B. Anzano M. A. Wakefield L. M. Roche N. S. Stern D. F. Sporn M. B. (1985). Type beta transforming growth factor: a bifunctional regulator of cellular growth. *Proc. Natl. Acad. Sci. U.S.A.* 82 119–123. 10.1073/pnas.82.1.119 3871521 PMC396983

[B97] Roman A. Munger K. (2013). The papillomavirus E7 proteins. *Virology* 445 138–168. 10.1016/j.virol.2013.04.013 23731972 PMC3783579

[B98] Rozenblatt-Rosen O. Deo R. C. Padi M. Adelmant G. Calderwood M. A. Rolland T. (2012). Interpreting cancer genomes using systematic host network perturbations by tumour virus proteins. *Nature* 487 491–495. 10.1038/nature11288 22810586 PMC3408847

[B99] Saint Just Ribeiro M. Wallberg A. E. (2009). Transcriptional mechanisms by the coregulator MAML1. *Curr. Protein Pept. Sci.* 10 570–576. 10.2174/138920309789630543 19751190

[B100] Schaper I. D. Marcuzzi G. P. Weissenborn S. J. Kasper H. U. Dries V. Smyth N. (2005). Development of skin tumors in mice transgenic for early genes of human papillomavirus type 8. *Cancer Res.* 65 1394–1400. 10.1158/0008-5472.CAN-04-3263 15735026

[B101] Schiffman M. Castle P. E. Jeronimo J. Rodriguez A. C. Wacholder S. (2007). Human papillomavirus and cervical cancer. *Lancet* 370 890–907. 10.1016/S0140-6736(07)61416-0 17826171

[B102] Schiller J. T. Lowy D. R. (2012). Understanding and learning from the success of prophylactic human papillomavirus vaccines. *Nat. Rev. Microbiol.* 10 681–692. 10.1038/nrmicro2872 22961341 PMC6309166

[B103] Siegel R. L. Miller K. D. Jemal A. (2018). Cancer statistics, 2018. *CA Cancer J. Clin.* 68 7–30. 10.3322/caac.21442 29313949

[B104] Smelov V. Hanisch R. Mckay-Chopin S. Sokolova O. Eklund C. Komyakov B. (2017). Prevalence of cutaneous beta and gamma human papillomaviruses in the anal canal of men who have sex with women. *Papillomavirus Res.* 3 66–72. 10.1016/j.pvr.2017.02.002 28720458 PMC5883282

[B105] South A. P. Purdie K. J. Watt S. A. Haldenby S. Den Breems N. Y. Dimon M. (2014). NOTCH1 mutations occur early during cutaneous squamous cell carcinogenesis. *J. Invest. Dermatol.* 134 2630–2638. 10.1038/jid.2014.154 24662767 PMC4753672

[B106] Stransky N. Egloff A. M. Tward A. D. Kostic A. D. Cibulskis K. Sivachenko A. (2011). The mutational landscape of head and neck squamous cell carcinoma. *Science* 333 1157–1160. 10.1126/science.1208130 21798893 PMC3415217

[B107] Tan M. J. White E. A. Sowa M. E. Harper J. W. Aster J. C. Howley P. M. (2012). Cutaneous beta-human papillomavirus E6 proteins bind Mastermind-like coactivators and repress Notch signaling. *Proc. Natl. Acad. Sci. U.S.A.* 109 E1473–E1480. 10.1073/pnas.1205991109 22547818 PMC3384212

[B108] Tommasino M. (2017). The biology of beta human papillomaviruses. *Virus Res.* 231 128–138. 10.1016/j.virusres.2016.11.013 27856220

[B109] Uberoi A. Lambert P. F. (2017). Rodent Papillomaviruses. *Viruses* 9:E362. 10.3390/v9120362 29186900 PMC5744137

[B110] Uberoi A. Yoshida S. Frazer I. H. Pitot H. C. Lambert P. F. (2016). Role of ultraviolet radiation in papillomavirus-induced disease. *PLoS Pathog.* 12:e1005664. 10.1371/journal.ppat.1005664 27244228 PMC4887022

[B111] Valcourt U. Carthy J. Okita Y. Alcaraz L. Kato M. Thuault S. (2016). Analysis of epithelial-mesenchymal transition induced by transforming growth factor beta. *Methods Mol. Biol.* 1344 147–181. 10.1007/978-1-4939-2966-5_9 26520123

[B112] Van Doorslaer K. Li Z. Xirasagar S. Maes P. Kaminsky D. Liou D. (2017). The papillomavirus episteme: a major update to the papillomavirus sequence database. *Nucleic Acids Res.* 45 D499–D506. 10.1093/nar/gkw879 28053164 PMC5210616

[B113] Vande Pol S. B. Klingelhutz A. J. (2013). Papillomavirus E6 oncoproteins. *Virology* 445 115–137. 10.1016/j.virol.2013.04.026 23711382 PMC3783570

[B114] Viarisio D. Gissmann L. Tommasino M. (2017). Human papillomaviruses and carcinogenesis: well-established and novel models. *Curr. Opin. Virol.* 26 56–62. 10.1016/j.coviro.2017.07.014 28778034

[B115] Viarisio D. Muller-Decker K. Accardi R. Robitaille A. Durst M. Beer K. (2018). Beta HPV38 oncoproteins act with a hit-and-run mechanism in ultraviolet radiation-induced skin carcinogenesis in mice. *PLoS Pathog.* 14:e1006783. 10.1371/journal.ppat.1006783 29324843 PMC5764406

[B116] Viarisio D. Mueller-Decker K. Kloz U. Aengeneyndt B. Kopp-Schneider A. Grone H. J. (2011). E6 and E7 from beta HPV38 cooperate with ultraviolet light in the development of actinic keratosis-like lesions and squamous cell carcinoma in mice. *PLoS Pathog.* 7:e1002125. 10.1371/journal.ppat.1002125 21779166 PMC3136451

[B117] Viarisio D. Muller-Decker K. Zanna P. Kloz U. Aengeneyndt B. Accardi R. (2016). Novel ß-HPV49 transgenic mouse model of upper digestive tract cancer. *Cancer Res.* 76 4216–4225. 10.1158/0008-5472.CAN-16-0370 27216183

[B118] Wallace N. A. Robinson K. Galloway D. A. (2014). Beta human papillomavirus E6 expression inhibits stabilization of p53 and increases tolerance of genomic instability. *J. Virol.* 88 6112–6127. 10.1128/JVI.03808-13 24648447 PMC4093859

[B119] Wallace N. A. Robinson K. Howie H. L. Galloway D. A. (2012). HPV 5 and 8 E6 abrogate ATR activity resulting in increased persistence of UVB induced DNA damage. *PLoS Pathog.* 8:e1002807. 10.1371/journal.ppat.1002807 22807682 PMC3395675

[B120] Wang D. Kanuma T. Mizunuma H. Takama F. Ibuki Y. Wake N. (2000). Analysis of specific gene mutations in the transforming growth factor-beta signal transduction pathway in human ovarian cancer. *Cancer Res.* 60 4507–4512. 10969799

[B121] Wang J. Zhou D. Prabhu A. Schlegel R. Yuan H. (2010). The canine papillomavirus and gamma HPV E7 proteins use an alternative domain to bind and destabilize the retinoblastoma protein. *PLoS Pathog.* 6:e1001089. 10.1371/journal.ppat.1001089 20824099 PMC2932728

[B122] Wang N. J. Sanborn Z. Arnett K. L. Bayston L. J. Liao W. Proby C. M. (2011). Loss-of-function mutations in Notch receptors in cutaneous and lung squamous cell carcinoma. *Proc. Natl. Acad. Sci. U.S.A.* 108 17761–17766. 10.1073/pnas.1114669108 22006338 PMC3203814

[B123] Wendel S. O. Wallace N. A. (2017). Loss of genome fidelity: Beta HPVs and the DNA damage response. *Front. Microbiol.* 8:2250. 10.3389/fmicb.2017.02250 29187845 PMC5694782

[B124] Weng A. P. Millholland J. M. Yashiro-Ohtani Y. Arcangeli M. L. Lau A. Wai C. (2006). c-Myc is an important direct target of Notch1 in T-cell acute lymphoblastic leukemia/lymphoma. *Genes Dev.* 20 2096–2109. 10.1101/gad.1450406 16847353 PMC1536060

[B125] White E. A. Howley P. M. (2013). Proteomic approaches to the study of papillomavirus-host interactions. *Virology* 435 57–69. 10.1016/j.virol.2012.09.046 23217616 PMC3522865

[B126] White E. A. Kramer R. E. Tan M. J. Hayes S. D. Harper J. W. Howley P. M. (2012a). Comprehensive analysis of host cellular interactions with human papillomavirus E6 proteins identifies new E6 binding partners and reflects viral diversity. *J. Virol.* 86 13174–13186. 10.1128/JVI.02172-12 23015706 PMC3503137

[B127] White E. A. Sowa M. E. Tan M. J. Jeudy S. Hayes S. D. Santha S. (2012b). Systematic identification of interactions between host cell proteins and E7 oncoproteins from diverse human papillomaviruses. *Proc. Natl. Acad. Sci. U.S.A.* 109 E260–E267. 10.1073/pnas.1116776109 22232672 PMC3277141

[B128] White E. A. Walther J. Javanbakht H. Howley P. M. (2014). Genus beta human papillomavirus E6 proteins vary in their effects on the transactivation of p53 target genes. *J. Virol.* 88 8201–8212. 10.1128/JVI.01197-14 24850740 PMC4135955

[B129] Wu L. Aster J. C. Blacklow S. C. Lake R. Artavanis-Tsakonas S. Griffin J. D. (2000). MAML1 a human homologue of Drosophila mastermind, is a transcriptional co-activator for NOTCH receptors. *Nat. Genet.* 26 484–489. 10.1038/82644 11101851

[B130] Xue X. Y. Majerciak V. Uberoi A. Kim B. H. Gotte D. Chen X. (2017). The full transcription map of mouse papillomavirus type 1 (MmuPV1) in mouse wart tissues. *PLoS Pathog.* 13:e1006715. 10.1371/journal.ppat.1006715 29176795 PMC5720830

[B131] Yamashita T. Segawa K. Fujinaga Y. Nishikawa T. Fujinaga K. (1993). Biological and biochemical activity of E7 genes of the cutaneous human papillomavirus type 5 and 8. *Oncogene* 8 2433–2441. 8395681

[B132] Zanier K. Charbonnier S. Sidi A. O. Mcewen A. G. Ferrario M. G. Poussin-Courmontagne P. (2013). Structural basis for hijacking of cellular LxxLL motifs by papillomavirus E6 oncoproteins. *Science* 339 694–698. 10.1126/science.1229934 23393263 PMC3899395

[B133] Zhang M. Biswas S. Qin X. Gong W. Deng W. Yu H. (2016). Does Notch play a tumor suppressor role across diverse squamous cell carcinomas? *Cancer Med.* 5 2048–2060. 10.1002/cam4.731 27228302 PMC4884632

